# Spatial technologies to evaluate the HIV-1 reservoir and its microenvironment in the lymph node

**DOI:** 10.1128/mbio.01909-24

**Published:** 2024-07-26

**Authors:** Fatima Zaman, Melissa L. Smith, Ashwin Balagopal, Christine M. Durand, Andrew D. Redd, Aaron A. R. Tobian

**Affiliations:** 1 Department of Pathology, Johns Hopkins University School of Medicine, Baltimore, Maryland, USA; 2 Department of Biochemistry and Molecular Genetics, University of Louisville School of Medicine, Louisville, Kentucky, USA; 3 Division of Infectious Diseases, Department of Medicine, Johns Hopkins University, Baltimore, Maryland, USA; 4 Laboratory of Immunoregulation, National Institute of Allergy and Infectious Diseases, National Institutes of Health, Bethesda, Maryland, USA; 5 Institute of Infectious Disease and Molecular Medicine, University of Cape Town, Cape Town, South Africa; Albert Einstein College of Medicine, Bronx, New York, USA

**Keywords:** human immunodeficiency virus, lymph node, tfh, HIV-1, spatial technologies, HIV-1 reservoir

## Abstract

The presence of the HIV-1 reservoir, a group of immune cells that contain intact, integrated, and replication-competent proviruses, is a major challenge to cure HIV-1. HIV-1 reservoir cells are largely unaffected by the cytopathic effects of viruses, antiviral immune responses, or antiretroviral therapy (ART). The HIV-1 reservoir is seeded early during HIV-1 infection and augmented during active viral replication. CD4+ T cells are the primary target for HIV-1 infection, and recent studies suggest that memory T follicular helper cells within the lymph node, more precisely in the B cell follicle, harbor integrated provirus, which contribute to viral rebound upon ART discontinuation. The B cell follicle, more specifically the germinal center, possesses a unique environment because of its distinct property of being partly immune privileged, potentially allowing HIV-1-infected cells within the lymph nodes to be protected from CD8+ T cells. This modified immune response in the germinal center of the follicle is potentially explained by the exclusion of CD8+ T cells and the presence of T regulatory cells at the junction of the follicle and extrafollicular region. The proviral makeup of HIV-1-infected cells is similar in lymph nodes and blood, suggesting trafficking between these compartments. Little is known about the cell-to-cell interactions, microenvironment of HIV-1-infected cells in the follicle, and trafficking between the lymph node follicle and other body compartments. Applying a spatiotemporal approach that integrates genomics, transcriptomics, and proteomics to investigate the HIV-1 reservoir and its neighboring cells in the lymph node has promising potential for informing HIV-1 cure efforts.

## INTRODUCTION

Globally, approximately 39 million individuals are infected with HIV, and almost one and a half million individuals were newly infected with the virus in 2021 (1). The mortality rate in most parts of the world has significantly decreased due to antiretroviral therapy (ART); however, ART fails to fully eradicate HIV-1 in the body due to the persistence of the HIV-1 viral reservoir (2).

The HIV-1 reservoir was first identified in resting memory CD4+ (rCD4) T cells in blood and lymph nodes (3, 4). The HIV-1 reservoir is defined as a group of cells that has two characteristics: first, they contain a replication-competent provirus, and second, the cells have stable kinetic properties over prolonged durations that allows the virus to persist (5). The HIV-1 reservoir is initially seeded early during the eclipse phase of infection, potentially before the virus appears in the circulation, and is maintained and augmented throughout viremia, and eventually decaying exceedingly slowly during ART (6–9).

The cell pool contributing to the HIV-1 reservoir demonstrates profound inter- and intra-cellular heterogeneity in phenotype, gene expression profile, and epigenetic status (10). The heterogeneous populations of cells comprising the HIV-1 reservoir are often classified by their memory status or effector function (11). Memory T cell types associated with the contribution to the HIV-1 reservoir include central memory T cells, effector memory T cells, transitional memory T cells, and memory T cells with stem cell-like properties(11–13). Effector function cell types that have been described to contribute to the HIV-1 reservoir include Th1, Th17, regulatory T cells (Tregs), and T follicular helper (Tfh) cells (11–13). Although we focus in this review on CD4+ T cells, other cell types, including macrophages, follicular dendritic cells (FDCs), monocytes, dendritic cells (DC), B cells, and natural killer (NK) cells, have also been described as potential cellular reservoirs for HIV-1 (5, 14). Multiple nonexclusive hypotheses have been proposed to explain the persistence and maintenance of the HIV-1 reservoir despite ART, and in general, they have two common themes (15). First, limited viral gene transcription and subsequent reduced expression help guard the infected cells from anti-HIV-1 immune responses, in particular cytotoxic CD8+ T cell and antibody responses. Second, the long-term stability of the HIV-1 reservoir can also be attributed to the long lifespan of the memory CD4+ T cell subset and its continuous renewal through clonal expansion (16). After activation and expansion in response to a foreign antigen, a portion of activated CD4+ T cells transition to a quiescent state with minimal active function. Upon re-exposure to their specific antigen, these memory CD4+ T cells re-expand rapidly. The HIV-1 reservoir exploits this memory phenotype by (i) persisting indefinitely and (ii) replenishing and augmenting through clonal expansion of the memory CD4+ T cell host (5).

The mechanisms that drive clonal expansion primarily include homeostatic proliferation, antigen-driven proliferation, and regulation by viral integration (17). Homeostatic proliferation occurs in the absence of cognate antigens and is driven by cytokines, including IL-7 and IL-15 (17, 18). Antigenic stimulation of the HIV-1 reservoir may also contribute to clonal expansion (18). Co-infection with chronic viruses, including Epstein-Barr virus (EBV) and cytomegalovirus, is thought to be associated with clonal expansion of the HIV-1 reservoir (17, 19–22).

The anatomical sites linked with the HIV-1 reservoir include the circulating immune cells, lymph nodes, gut-associated lymphoid tissue (GALT), tonsils, spleen, and the central nervous system (23). The frequency and proviral makeup of latently infected cells are similar in blood and lymph nodes, suggesting trafficking between these compartments and other tissues in the body (4, 24, 25). Lymph nodes and GALT have been attributed as the main contributors to cellular sources of viral rebound in virally suppressed individuals (26).

The lymph node is a complex organ with multiple well-characterized microenvironments. A better understanding of the HIV-1 reservoir found within the lymph node is essential for fully understanding seeding, persistence, and viral rebound. In this review, we discuss the lymph node and, more precisely, the role of B cell follicles and germinal centers in the persistence of the HIV-1 reservoir. We also discuss how spatial multi-omics technology may be able to advance our understanding of the HIV-1 reservoir in the lymph node.

## LYMPH NODE

**Fig 1:**
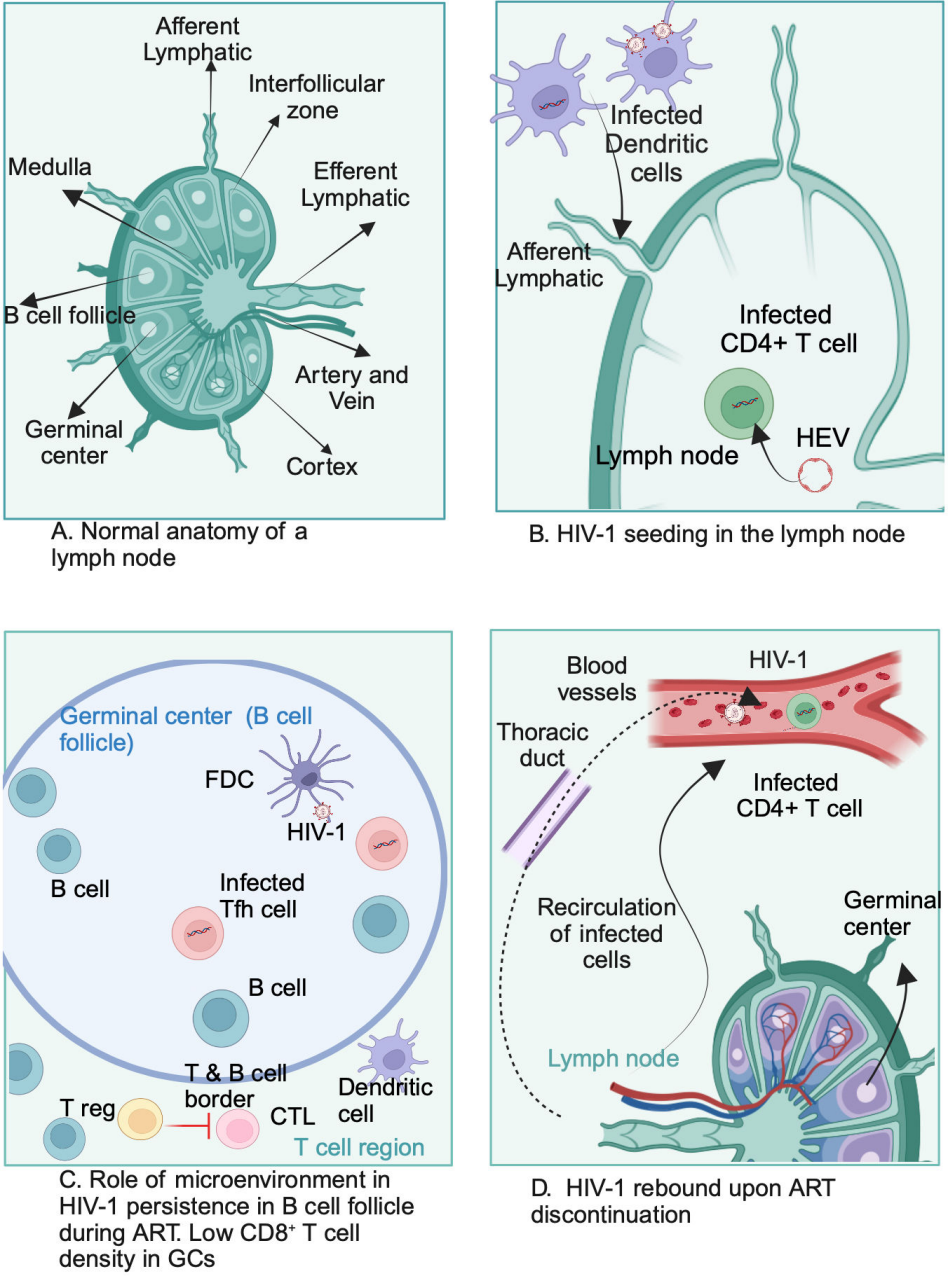
Potential mechanism of HIV-1 reservoir persistence and rebound in the lymph node. Schematic diagram of a lymph node illustrates HIV-1-infected Tfh cells and the cellular microenvironment inside the lymph node that supports establishing and maintaining the HIV-1 reservoir. Abbreviations: Tfh, T follicular helper cell; HEV, high endothelial venule; CTL, cytotoxic T lymphocyte; FDC, follicular dendritic cell. (A). Shown is a normal anatomy of the lymph node. (B) Infected CD4+ T cells and infected dendritic cells trafficking to the lymph nodes through afferent lymphatic or HEV. (C) Uninfected Tfh cells can get infected in the follicle via FDC or outside the follicle via dendritic cells. (D) Potentially, the infected CD4+ T cell can recirculate from the follicles to the body via efferent lymphatics or draining blood vessels. Created with BioRender.com.

Lymph nodes are vascularized secondary lymphoid organs characterized by a distinct architecture and compartmentalization of cell types (Fig. 1). They are interconnected by a network of lymphatic vessels that carry extracellular fluid from tissues to the lymph node and eventually circulate back into the blood (27, 28). Soluble and DC-associated antigens are filtered in the lymph node through afferent lymphatics, and these DCs and macrophages present antigens to the lymphocytes (Fig. 1). Lymph nodes also consist of aggregate lymphocytes surrounded by non-leukocyte cells that provide a structural framework. The outer cortex contains B cell follicles, which consist primarily of B cells. T cells are diffusely located around the B cell follicle in the paracortex. The location of B and T cells may fluctuate upon activation, and lymphocytes may migrate to the border of the follicle. Upon activation, some B cell follicles develop germinal centers where B cells undergo proliferation and differentiation into plasma cells with T cell help (27, 28).

### Germinal centers

Each germinal center contains two zones: a dark zone and a light zone. The dark zone consists of centroblasts, which are proliferating B cells, and the light zone contains centrocytes, which are B cells that have stopped proliferating and undergoing selection for survival and further maturation. Tfh and FDCs are also found in the light zone. Tfh cells play a critical role in B cell selection and differentiation, whereas FDCs provide a structural meshwork around which the germinal centers are formed (27, 28).

Germinal centers have generated great interest as sites of HIV-1 reservoir seeding, persistence, and viral rebound upon ART discontinuation (26, 29–33). The cellular components of the germinal center, Tfh cells and FDCs, contribute to the viral reservoir. It has been suggested that the Tfh cell population makes up a considerable proportion of CD4+ T cells that harbor HIV-1 DNA in the lymph node (30). HIV-1 infection induces Tfh expansion in the germinal center; a similar expansion is not seen outside this region (34). Tfh precursor cells can be infected by HIV-1-infected lymph node dendritic cells in the T cell zone or after differentiation within the germinal center upon exposure to extracellular HIV-1 virion trapped on FDCs (Fig. 1) (35–37). The precise mechanisms of HIV-1 infection of Tfh cells by dendritic cells or FDCs remain poorly understood (Table 1) (38).

**TABLE 1:** Central knowledge gaps and potential research questions on the HIV-1 reservoir in the lymph node**^*a*^**

Heterogeneity of the HIV-1 reservoir
Characterization of Tfh cell subset that contributes to the HIV-1 reservoir
Timing and mechanism of Tfh cell infection
Plasticity and fate of Tfh cell pre- and post-HIV infection
Cellular microenvironment of the HIV-1 reservoir
Spatial organization of the HIV-1 reservoir and its neighboring cell
Mechanism of clonal expansion of the HIV-1 reservoir
Distinct pockets of clonally expanded HIV-1 reservoir
Movement of the clonal expanded HIV-1 reservoir cells among different regions of the lymph node

^*a*^ Abbreviations: Tfh, T follicular helper cell.

Tfh cells demonstrate heterogenous expression of C-C chemokine receptor 5 (CCR5), the primary co-receptor for HIV-1 entry (38, 39). Three distinct hypotheses have been proposed to explain the mechanism of Tfh cell infection by HIV-1 despite heterogenous expression of CCR5. The first hypothesis is that there is a subset of Tfh cells that express CCR5 at high levels and are consequently susceptible to HIV infection (38). In contrast, those that have reduced expression of co-receptors have low susceptibility. An alternative hypothesis proposes a temporal change in the expression of the co-receptor: high CCR5-expressing Tfh cells are infected with HIV-1 in the T cell zone, and post-infection, CCR5 is downregulated while Tfh cells migrate to the germinal center. A third hypothesis that explains reduced CCR5 expression on Tfh is that CXCR3 serves as an alternate co-receptor for HIV-1 entry into Tfh cells (34).

In normal physiology, Tfh cells play a critical role in B cell differentiation and affinity maturation. However, during chronic HIV-1 infection, dysregulation of Tfh cell responses has been observed. The dysfunction in Tfh cells leads to impaired T cell-dependent B cell responses and subsequently extensive defects in the humoral arm of the immune system (40, 41). The cause of this dysfunction is thought to be an imbalance between T-helper 1(Th1) and T-helper 2(Th2) subsets of Tfh cells and the expression of immune checkpoint molecules, such as PD-1/PD-L1 (40, 41). PD-L1 expressed by germinal center B cells or by PD-L1-positive virions captured by FDC can potentially interact with PD-1 on Tfh cells and lead to impaired humoral responses despite an apparent increase in Tfh cells in the germinal center (40, 42–45). Suboptimal antibody responses can also partially be attributed to the reduced ability of HIV-1-specific B cells to enter and/or remain in the germinal center during chronic HIV-1 infection (46). Alongside Tfh cells, FDCs found in the germinal center and the lymphoreticular network of FDCs can trap and maintain HIV-1 particles on their surface and may facilitate viral transmission (35, 47).

### Lymph node follicle

Cytotoxic CD8+ T cells are essential in host defense against intracellular viruses through cytolytic and cytokine-mediated mechanisms. Elite controllers who suppress viral replication without ART often have a higher frequency of HIV-1-specific CD8+ T cells that preferentially home to B cell follicles; conversely, a similar distribution is not observed in chronic progressors (48). Although these CD8+ T cells have reduced cytolytic activities, they can potentially control infection through cytotoxic-independent mechanisms, including secretion of soluble factors such as cytokine secretions (48–50).

For effective killing of HIV-1 reservoir components by CD8+ T cells, these cells must be near the cells comprising the HIV-1 reservoir. However, it has been observed that CD8+ T cells are infrequently present in the follicle and almost absent in the germinal center, and subsequently, there is reduced interaction with the HIV-1 reservoir (51). As discussed earlier, HIV-1 is concentrated in the B cell follicle, where there is a considerably lower frequency of CD8+ T cells (Fig. 1). The low density of CD8+ T cells has been attributed to reduced expression of CXCR5, the essential homing receptor for the germinal center (52). In an acute simian immunodeficiency virus (SIV) infection model, virus-specific CD8+ T cells are mostly excluded from the germinal center (53). It has been proposed that delayed recruitment of virus-specific CD8+ T cells to the follicle during the early stage of infection may facilitate HIV-1 persistence in the germinal center. In addition to reduced homing to the follicle, CD8+ T cells in the follicle have impaired cytolytic activities and cellular differentiation, potentially due to impaired transcription (54).

NK cells are also an important arm of the antiviral host immune response, but unlike CD8+ T cells and other antigen-specific cells, NK cells don’t require prior priming by an antigen. An inverse relationship between the quantities of CXCR5+ NK cells in the follicle and HIV-1 viral burden in the lymph nodes has been observed (55). Similar to follicular CD8+ T cells, NK cells are infrequently found in the follicle and show reduced expression of cytolytic molecules; nonetheless, CD8+ T cells and NK cells hold the potential to control HIV-1 persistence through non-cytolytic cellular activities (55).

### Junction of B and T cell zone at the border of the follicle

Tregs are a subset of CD4+ T cells characterized by their immune-suppressing function. The HIV-1 reservoir potentially exploits this intrinsic property of Tregs to maintain self-tolerance, facilitating HIV persistence. Tregs within the lymph node are located at the T and B cell borders outside the B cell follicle (56, 57). Their unique location allows them to exert regulatory functions by suppressing CD8+ T cells from entering the follicle and, subsequently, the germinal center, potentially preventing cytotoxic HIV-1 reservoir clearance in these regions. Tregs also secrete the inhibitory cytokine, IL-10, which may also act to maintain the HIV-1 reservoir and, subsequently, HIV persistence. In an SIV model, virus-containing cells were found near cells expressing IL-10, suggesting that the secretory function of Tregs supports the homing of the HIV-1 reservoir in the follicle (58).

In addition to conventional Tregs, a unique subset of regulatory T cells called T follicular regulatory cells are observed in the lymph node (57, 59). The phenotypic and functional properties of T follicular regulatory cells overlap with Tregs and Tfh cells. Like Tregs, T follicular regulatory cells are located at the B and T cell borders and are seldom observed in the germinal center. Their suppressive function blocks entry of CD8+ T cells into the germinal center from outside the follicle (57, 59–61). Their unique location also allows them to moderate and/or inhibit Tfh cell and B cell interactions, resulting in suboptimal humoral responses during HIV-1 infection. As their name implies, T follicular regulatory cells also share properties with Tfh cells. Despite ongoing interest, studying T follicular regulatory cells remains challenging as the main surface markers used to identify T follicular regulatory cells are also expressed by Tfh cells or conventional Tregs (60).

### HIV-1-infected cells vs. HIV-1 reservoir in the lymph node

HIV-infected cells are complex and include various types of cells including provirus that are intact, defective, or hypermutated. Characterizing HIV-1-infected cells that persist despite ART and lead to rebound viremia are essential in developing HIV-1 cure (62). The majority of intact proviral sequences in the lymph node are harbored by CXCR5+, CXCR5−PD-1+, and CXCR5−PD-1− CD4 T cells (62). Focal enrichment is HIV-1 reservoir cells exhibited in TFH cells and CD4+ tissue-resident memory T phenotypes, and these HIV-1 reservoir cells express phenotypic characteristics of survival and apoptosis resistance (63). Dendritic cells in the lymph node also contribute to the HIV-1 reservoir as they contain intact and replication-component viruses (37).

### Knowledge gaps  

#### Cellular composition and heterogeneity of the HIV-1 reservoir

Despite the widely accepted role of Tfh in HIV-1 persistence, the precise subset of Tfh cells involved remains unclear (Table 1). Tfh cells consist of a heterogeneous and distinct population of cell subtypes classified by the expression of cell surface receptors associated with T helper lineages, CXCR3 (Th1), CCR4 (Th2), and CCR6 (Th17) (64). Some evidence suggests that blood memory CXCR3+ CD4 T cells are enriched in cells containing inducible replication competent virus. CXCR3+ Tfh cells have an increased capacity to enter the circulation compared with their CXCR3− counterpart, implying that memory CXCR3+ cells in blood may have originated from the HIV-1 reservoir in the lymph node (65, 66). However, these findings need to be explored further.

In addition to functional subsets of Tfh, the presence of memory subtypes further complicates the understanding of their role in HIV persistence. The HIV-1 reservoir may display central memory, effector memory, and transitional memory properties (11, 13, 67). The plasticity of Tfh cells additionally complicates the understanding of heterogeneous functional and memory subsets of Tfh cells. Consequently, the precise subset of Tfh cells contributing to the HIV-1 reservoir and its phenotypic markers and fate are not well established.

#### Clonal expansion of the HIV-1 reservoir

Due to clonal expansion, it is possible that HIV-1 cells may be found within discrete clusters or microfoci in the lymph node, with the circulation then acting to distribute clonally expanded cells from the lymph node to peripheral organ tissues. Much remains to be done to thoroughly explore this hypothesis, including the development of robust methods to study tissue-associated latently infected cells.   

#### Cellular microenvironment of the HIV-1 reservoir

The cellular microenvironment of the HIV-1 reservoir may promote its persistence and maintenance. However, little is known of the viral and host mechanisms that induce an immunoprotected/sequestered microenvironment. Immune exhaustion is associated with chronic HIV persistence (68–70). It is suggested that immune checkpoint molecules limit cytotoxic T cell activity, thus protecting the HIV-1 reservoir from immune-mediated cell lysis (68–71). However, the role of immune checkpoint molecules and their ligands for seeding and maintaining the HIV-1 reservoir must be evaluated. Defining these pathways would permit exploring the potential therapeutic role of immune checkpoint blockade in reversing CD4 HIV-1 latency as well reversing exhaustion of HIV-1-specific CD8 T cells (72).

#### Temporal shift of the HIV-1 reservoir between T and B cell zone

The paucity of CD8+ T cells in the lymphoid follicle likely provides a more favorable environment for HIV-1 reservoir maintenance in the germinal center than the extrafollicular region (53). However, given the relative immune-sequestered nature of the follicle, it needs to be explored whether HIV-infected cells can shift from the extrafollicular zone during early infection to the B cell zone of the lymph node during chronic infection. Interestingly, a recent study demonstrates a higher abundance of SIV RNA in the T cell zone compared with the B cell zone after ART interruption (25). However, in contrast, in the human lymph node and GALT study, rebounding HIV-1 variants were detected first in the B-cell follicles upon treatment interruption (26). Hence, the spatial origin of these SIV RNA-containing cells needs to be assessed to confirm the temporal shift from the extrafollicular zone to the B cell follicle (25).The temporal shift of HIV-1 reservoir cells between zones may be associated with diminished sensitivity of these cells to CD8+ T cell killing; an enhanced understanding of the temporal shift is needed to understand the mechanism of the HIV-1 reservoir seeding in the lymph node.

#### Spatial context of the HIV-1 reservoir and its neighboring immune cell 

Traditional methods used to study the HIV-1 reservoir in the lymph node have thus far overlooked the spatial and temporal context. They largely fail to demonstrate the localization of the HIV-1 reservoir and the relative organization of immune cells, limiting our understanding of the interaction between the HIV-1 reservoir and the surrounding immune microenvironment and the extracellular matrix (Table 1). A comprehensive knowledge of the HIV-1 reservoir and its neighboring cells within lymph nodes provide information of HIV-1 persistence despite prolonged ART, and future studies should incorporate spatial methods to investigate the HIV-1 reservoir.

### Spatial multi-omics

#### Spatial proteomics

Distinct cell types arrange and operate together to form tissues and organs. Spatial characterization of these cells is essential for understanding cell-to-cell communication and anatomical organization. Traditional immunohistochemistry (IHC) methods can only detect single targets in a tissue, and conventional immunofluorescence (IF)-based methods can detect up to four targets in a single section. With the advancement in targeted spatial approaches in medicine, traditional methods do not fully meet the needs for characterizing the HIV-1 reservoir or its neighboring cells within intact tissue or structured organ environments. Advanced multiplexed protein detection methods that have a higher capacity to simultaneously detect multiple targets in a given tissue have been developed. The common theme between these methods is the utilization of primary antibodies against the protein of interest. However, these technologies vary widely in target detection sensitivity, specific targets for which reagents are available, and the instrumentation needed.

##### Iterative antibody staining

The iterative multi-cycle antibody method increases detection target capacity within the tissue sections compared with more traditional methods such as IHC or IF. This method is based on sequential staining followed by scanning and washing of the antibodies targeted to the protein of interest (73). It is compatible with both IHC- and IF-based detection methods. This method is commonly used to detect seven to nine targets. However, newer technologies such as iterative bleaching can extend multiplex potential to detect more than 65 parameters (74). Benefits of using the iterative antibody staining method over others include that it can be performed using commonly available reagents and scanned using regular microscopes, requiring no single-use specialized equipment. However, this method is mostly performed manually and can be extremely labor intensive.

##### Multiplexed signal amplification

Multiplexed signal amplification methods can detect multiple biomarkers and are beneficial for the detection of lowly expressed antigens. An example of multiplexed signal amplification is Opal (Akoya Biosciences, Marlborough, MA) (73). Opal uses tyramide signal amplification and is similar to the iterative antibody staining method in its use of sequential staining and antibody stripping. However, in this case, the secondary antibody is conjugated with a horseradish peroxidase that activates the tyramide-fluorophore complex. The activated tyramide-fluorophore complex is deposited, and antibodies are washed away. Targets are identified based on fluorophores linked to tyramide and scanned together at the end, removing the need for iterative scanning. Opal can detect up to nine targets, including a nuclear stain. Multiplexed signal amplification generally works well with formalin-fixed paraffin-embedded tissues but has limited application for fresh-frozen tissue sections.

##### DNA-barcoded antibodies

For DNA-barcoded antibody-based methods, antibodies are labeled with DNA oligonucleotides instead of fluorophores (75). By applying this method, the phenoCycler (Akoya Biosciences, Marlborough, MA) and GeoMx digital spatial profiler (NanoString, Seattle, WA) generate multiplexed spatial proteomic data. With the PhenoCycler, a tissue is stained with a panel of DNA-barcoded antibodies in a single step. Three fluorescently tagged reporters complementary to the barcode of the antibodies are dispensed on the stained tissue (75). Subsequently, the tissue is scanned by the microscope integrated into the PhenoCycler. After scanning, the reporters are removed through a gentle isothermal wash (75). Through sequential binding and washing out of fluorescent-tagged reporters, expression and distribution of >100 targets can be captured (75). Similarly, GeoMx DSP uses target-specific antibodies conjugated with photocleavable oligonucleotides. In GeoMx profiling, the tissue is stained with three or four DNA-barcoded antibodies in order to select the region of interest (ROI). Within the ROI, oligonucleotides are then released from their respective antibodies using UV light (76). Released oligonucleotides are counted and sequenced to evaluate expression. GeoMx can evaluate up to 570+ markers per ROI (76). In addition to protein expression, GeoMx can also detect gene expression through DNA barcoding-based methods (76). Together, DNA barcoding-based methods have advanced the field by dramatically increasing multiplexing capacity. However, these technologies are still relatively new in the field, and so, there are few supporting publications for this technology, but they hold the potential to provide invaluable information on cellular microenvironment within the tissue.

##### Ionizable metal mass tagging

Another alternate approach to overcome the limitations of the fluorophore-based approach is to utilize metal reporter-tagged antibodies. Metal reporter-tagged antibodies are identified and quantified through mass spectrometry. Metal isotopes have minimal overlap, and >40 targets can be simultaneously detected in single cells. Imaging mass cytometry (Standard BioTools, South San Francisco, CA) and multiplex ion beam imaging (Ionpath, Menlo Park, CA) apply metal mass tag-based methods to provide spatial proteomic information (73). In imaging mass cytometry, tissues are labeled with metal-tagged antibodies, followed by spot by spot and line by line laser beam ablation (77). The released metal reporter from the target is ionized and detected by mass cytometry (77). This method permits imaging of up to 32 proteins at a subcellular resolution (77). Multiplex ion beam imaging is similar to imaging mass cytometry, but it utilizes secondary antibodies tagged with metal reporters. It can analyze up to 100 targets simultaneously (73). Nevertheless, an increased level of multiplexity is accompanied with constraints in cost and time (78).

### Spatial transcriptomics

Spatial transcriptomics (ST) identifies gene expression by measuring mRNA with or without parallel protein detection at a cellular and/or subcellular level in structurally intact tissue sections. ST technologies have been applied to study spatial gene expression among various cell types and their cellular neighborhoods (79). The various available ST technologies are based on either sequencing or hybridization methods for detection and are reviewed below.

#### *In situ* hybridization

*In situ* hybridization-based spatial technologies are based on single-molecule Fluorescence *in situ* hybridization (smFISH) technology and involve complementary hybridization of fluorescent probes to RNA molecules followed by scanning the probes using fluorescence microscopy. smFISH has been modified to enable highly multiplexed detection with the advancements of mixing colors into pseudo colors, combinatorial barcoding, and sequential imaging rounds. In seqFISH+, up to 10,000 genes can be visualized per sample, and their expression quantified at a subcellular level in intact tissue utilizing pseudo colors for detection (80). Like seqFISH+, multiplex error-robust fluorescence *in situ* hybridization (MERFISH, Vizgen, Cambridge) can detect ~10,000 genes at the subcellular level but with higher capture efficiency than seqFISH+ due to its use of sequential rounds of hybridizations, followed by image decoding using a novel combinatorial barcoding system with robust error correction (81). Modifying hybridization-based technologies has successfully increased the number of genes analyzed at the subcellular level; however, genes are enriched by an a priori-targeted panel. Further advancement is needed to enable these technologies to perform transcriptome-wide profiling (82).

#### *In situ* sequencing

In the *in situ* sequencing-based method, mRNA transcripts are sequenced and read out within the intact tissue. In this method, transcripts can be first reverse transcribed into cDNA by priming with random hexamer or oligo-(dT) primers, followed by signal amplification and subsequent sequencing (81, 83–85). Signal amplification of the cDNA is performed generally by rolling circle amplification. The resulting product of rolling cycle amplification is subjected to either sequencing-by-ligation or sequencing-by-synthesis (86). In sequencing-by-ligation, first. an anchor primer binds to a targeted sequence followed by hybridization of interrogating probes. Interrogating probes are made up nucleotide basses and consist of four libraries with each with each library labeled with distinct fluorophore. The interrogating probe that best matches hybridizes with its fluorophore, and the sample is imaged (87). The method based on sequencing-by-synthesis involves incorporation of fluorescent-conjugated oligonucleotide (86). Like *in situ* hybridization-based technologies, *in situ* sequencing also provides subcellular resolution. *In situ* sequencing-based technologies, including STARMap, are primarily targeted and use custom probes that hybridize with intracellular RNA; however, FISSEQ and its latter adaptation, ExSeq, are untargeted and use adapter sequence-tagged random hexamers (83, 88, 89).

In general, *in situ* sequencing can be challenging to replicate outside the original laboratory; however, 10× Genomics has commercialized a version of this method in their Xenium *in situ* pipeline, which is based on probe-assisted sequencing of a targeted panel (90). Briefly, tissue sections are mounted on the Xenium slides, and probes with the spatial barcodes located on the slides hybridize with the targeted RNA transcripts in the tissue (90). Bound probes are ligated, forming a circular template optimal for rolling circle amplification of the probe with a unique barcode (90). The amplified probe products are hybridized with fluorescent probes followed by imaging and decoding. This is repeated in series of cycles, and image data processing allows identification and spatial location of each targeted transcript (14).

#### *In situ* capturing

*In situ* capturing-based method captures the transcripts *in situ* from intact tissue, followed by sequencing *ex vivo*. The most popular spatial transcriptomic technology, Visium (10× Genomics, Pleasanton, CA), is based on this method. In the preparation of fresh-frozen tissue, arrays of location-specific poly-T oligonucleotides are laid on a slide with oligonucleotide-tagged spots. The oligo-dT priming allows for the capture of all transcripts that contain a poly-A tail, including all mRNA transcripts. Visium offers a resolution of 50 to 200 µm, which contains 1 to 10 cells in each spot depending on cell size. *In situ* capturing-based technologies provide transcriptome-wide analysis. Importantly, additional protocols, such as CITE-seq, have been incorporated into Visium workflows, allowing for oligonucleotide-labeled antibodies to be incorporated into the spatial *in situ* capture, providing both RNA and protein profiling from the same tissue section. In addition, further advancements of *in situ* capturing-based technologies including new methodologies, such as Slide-seq, PIXEL-seq, and Seq-scope appear to provide higher resolution (91, 92).

#### Considerations for selecting spatial omics technologies

Spatial ’omics methods include a variety of strategies for capturing protein and/or RNA in intact tissue; identifying the method that fits best depends on the research question and resources. Methods focusing on transcriptome-wide detection are better suited for hypothesis generation, whereas a more targeted approach may be taken for studying specific genes. Other considerations include tissue area, number of specimens, capture efficiency, and resolution. Importantly, tools that link spatial transcriptomics with protein detection are required to fully map the HIV-1 reservoir in the lymph node. An added challenge is to integrate *in situ* detection of proviral HIV-1 DNA with transcriptomic and proteomic mapping of host factors in conjunction with HIV-1 reservoir in the lymph node.

#### Spatial technologies in HIV-1/SIV-based lymph node research

To our knowledge, there is limited literature available on multi-omic spatial technologies in HIV-1/SIV-infected lymph nodes. Two unpublished studies have performed spatial transcriptomics of HIV+ lymph nodes using GeoMx or Visium, and one study looked at SIV+ lymph nodes through GeoMx and CosMx (93–95). In HIV-1-infected lymph nodes, germinal center HIV-1 reservoir identified through p24 expression showed a unique immune transcriptional profile and significantly upregulated HLA, MHC, CD40, and CD40L expression compared with uninfected germinal centers (94). In another study on HIV-1-infected lymph nodes, pro-inflammatory T cells within the HIV-infected lymph node were compared with the HIV-1-uninfected lymph node within the T cell zone (95). In SIV-infected lymph nodes, genetic profile differences are observed between regions infected with SIV vs. uninfected regions. Infected cellular neighborhoods showed altered immune pathways of B Cell Development, HLA signaling IL-4, I-17, and interferon (93).

## CONCLUSIONS

Although bulk or single-cell technologies have improved our understanding of HIV-1 pathology in the lymph node, many critical questions remain unanswered, especially regarding the microenvironment of the HIV-1 reservoir in lymph tissues that facilitate its persistence despite ART. Integrating spatial genomic, transcriptomic, and proteomic data to map HIV-1 reservoir will be powerful methods to disentangle the cellular and molecular characteristics that maintain HIV-1 reservoir. A successful HIV cure strategy that requires disrupting the permissive microenvironment of HIV-1 reservoir hinges on mapping HIV-1-infected lymphoid tissues to evaluate efficacy and mechanisms.
